# Type 2 diabetes as a major risk factor for COVID-19 severity: a meta-analysis

**DOI:** 10.20945/2359-3997000000256

**Published:** 2020-06-05

**Authors:** Lana C. Pinto, Marcello C. Bertoluci

**Affiliations:** 1 Departamento de Emergência Hospital de Clínicas de Porto Alegre Universidade Federal do Rio Grande do Sul Porto Alegre RS Brasil Departamento de Emergência, Hospital de Clínicas de Porto Alegre, Universidade Federal do Rio Grande do Sul, Porto Alegre, RS, Brasil; 2 Serviço de Endocrinologia Hospital de Clínicas de Porto Alegre Universidade Federal do Rio Grande do Sul Porto Alegre RS Brasil Serviço de Endocrinologia, Hospital de Clínicas de Porto Alegre, Universidade Federal do Rio Grande do Sul, Porto Alegre, RS, Brasil

**DEAR EDITOR,**

Coronavirus disease 2019 (COVID-19) has recently emerged as a rapidly spreading disease, affecting more than 100 countries worldwide and reaching pandemic proportions. The severity of COVID-19 ranges from a mild, self-limiting flu-like illness to a devastating pneumonia culminating in respiratory failure and death. Individuals with diabetes are particularly vulnerable to some respiratory viral infections, such as influenza A (H1N1) infection ( 1 ), the severe acute respiratory syndrome (SARS) ( 2 ), and the Middle East respiratory syndrome (MERS) ( 3 ). A higher mortality rate was recently suggested in patients with COVID-19 who had preexisting diabetes ( 4 ); according to the Chinese Centers for Disease Control and Prevention, COVID-19 case-fatality rates in patients with diabetes were around 7.3%, versus 2.3% in the general Chinese population.

**Figure 1:**
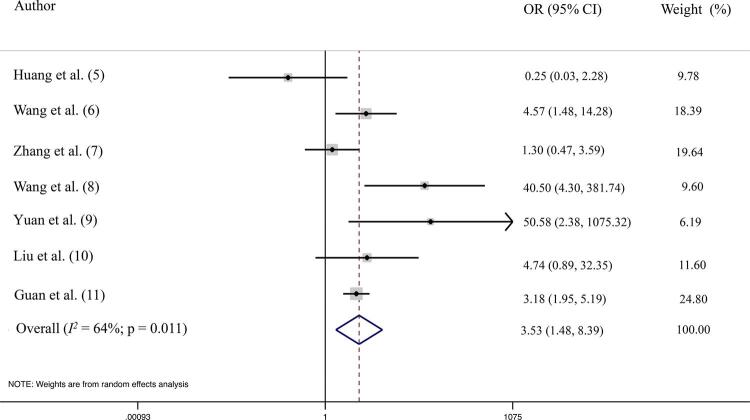
Forest-plot showing association of diabetes and severity of COVID-19, obtained from7descriptive studies.

We aimed to investigate the magnitude of this risk and its dependency on age. We performed a systematic search and meta-analysis for clinical reports of COVID-19 infection which included detailed descriptions of patients’ clinical profile – specifically, reporting information about the presence of diabetes at admission. The search strategy included the terms “clinical characteristics” AND “diabetes” AND “COVID-19” OR “SARS COV2” OR “coronavirus” OR “2019 n-Cov”, and yielded 7 records, all of them case series from China. The clinical status at admission was divided into severe (requiring intensive care or having an oxygen saturation <90%) or mild-to-moderate (not requiring ICU or oxygen saturation >90%). The meta-analysis included a total of 1592 patients, 138 with a previous diagnosis of diabetes and 1454 without diabetes. Among those with diabetes, 59 (42.75%) developed severe COVID-19 compared to 256 (17.60%) of non-T2DM patients, resulting in an odds ratio of 3.53 (95% confidence interval 1.48 to 8.39; *I* ^2^ 64%; p for heterogeneity = 0.011) ( Figure 1 ). The high heterogeneity of this analysis suggests that other factors could nonetheless be involved in the higher risk of this population. To address this issue, we performed a random meta-analysis with meta-regression using the mean age of patients as a covariate; there was no impact on our initial results.

Diabetes mellitus appears to be a major, age-independent risk factor for severity of COVID-19. Further studies are necessary to address mechanisms by which diabetes may affect the prognosis of COVID-19 and how improving glycemic control might impact the course of the disease.
